# Development and Application of a Novel ‘Green’ Antibacterial Black Garlic (*Allium sativum*)-Based Nanogel in Epidermal Wound Healing^§^

**DOI:** 10.17113/ftb.63.02.25.8873

**Published:** 2025-06

**Authors:** Mariah Sadaf, Anamika Das, Satadal Das, Subhankar Saha, Ketousetuo Kuotsu, Paramita Bhattacharjee

**Affiliations:** 1 Department of Food Technology and Biochemical Engineering, Faculty of Engineering and Technology, Jadavpur University, 700032 Kolkata, India; 2 School of Bio-Science and Engineering, Faculty of Interdisciplinary Science and Technology, Jadavpur University, 700032 Kolkata, India; 3 Department of Microbiology, Peerless Hospital & B. K. Roy Research Centre, 70009 Kolkata 4, India; 4 Department of Pharmaceutical Technology, Faculty of Engineering and Technology, Jadavpur University, 700032 Kolkata, India

**Keywords:** black garlic, alliin-rich extract, skin pathogens, epidermal wound healing, antibacterial topical nanogel

## Abstract

**Research background:**

Black garlic has been reported to have several health-promoting properties compared to fresh, raw garlic. The enzyme alliinase, which converts alliin to allicin, is deactivated at moderately high temperature, thus stripping away the typical pungent odour of fresh garlic during fermentation and rendering black garlic devoid of the typical garlic-like smell. To date, the antimicrobial activity of alliin-rich extract obtained from black garlic powder has not been reported. The objectives of this study are to explore the antibacterial/antifungal activity of alliin-rich black garlic extract against *Staphylococcus aureus*, *Escherichia coli* and *Candida albicans*, and to formulate a topical drug, based on the efficacy of the extract, using non-toxic, green ingredients in the form of a nanogel with promising wound-healing property and safe for human use.

**Experimental approach:**

Authenticated fresh garlic (*Allium sativum*) cloves were first fermented to yield black garlic. After fermentation, the brownish-black garlic cloves were peeled and ground into powder. The alliin-rich extract was then obtained by Soxhlet extraction. Nanogels were formulated using the alliin-rich extract and were subjected to a kinetic study of *in vitro* release. The antibacterial potency of the nanogels was also evaluated against *Staphylococcus aureus* (ATCC 29213) and *Escherichia coli* (ATCC 25922 and their multiple drug-resistant strains), followed by a skin irritation study on New Zealand albino rabbits.

**Results and conclusions:**

Soxhlet extraction of pulverized black garlic cloves using distilled water yielded an alliin-rich extract (6.4 mg/100 g garlic), which also contained additional bioactive organosulfur compounds with no reported toxicity. The antimicrobial potency (in terms of its minimum inhibitory concentration (MIC)) of the extract was evaluated against potent skin pathogens and was found to be ~15 µg/mL. The nanogels formulated with the alliin-rich extract showed shear thinning rheology and admirable sensory properties when tested by a panel. The *in vitro* release kinetic study showed a burst release of alliin (75 % of its content) from either gel within 5 min. Following a skin irritation test performed on male New Zealand albino rabbits, no clinical signs of toxicity/mortality, redness or swelling were observed in the animals. The nanogels applied individually on the epidermal wounds prevented external infection and accelerated wound healing from day 2 onwards, achieving complete healing by day 7. Moreover, the gel containing 4 % extract did not leave a scar on the wounded area after complete healing on day 7, establishing it as a promising topical antibacterial nanogel with accelerated epidermal wound-healing property, compared to a commercial broad-spectrum topical gel, used as a positive control.

**Novelty and scientific contribution:**

This study is the first to report on a newly developed ‘green’ nanogel containing antimicrobial bioactive compounds, namely, organosulfur compounds (diallyl disulfide, diallyl trisulfide, methyl-allyl-disulfide and methyl-allyl-trisulfide). The nanogel showed promising epidermal wound-healing properties and is therefore promising in clinical applications against common and potent human skin pathogens.

## INTRODUCTION

The underground bulb of fresh, raw garlic (*Allium sativum*) is consumed by humans as a spice and medicine (*1*, *2*). It contains several bioactive components (*3*) that confer strong antibacterial, antifungal, anti-inflammatory and anti-allergic properties (*4*, *5*). The key bioactive compounds of raw garlic include organosulfur compounds, such as diallyl thiosulfonate (allicin), diallyl sulfide (DAS), diallyl disulfide (DADS), diallyl trisulfide (DATS), ajoene, s-allyl-l-cysteine and alliin (*6*). Garlic cloves naturally contain alliin and also the enzyme alliinase. When garlic is crushed, alliinase converts alliin into allicin, which is responsible for the characteristic pungent odour of garlic (*7*, *8*).

Fresh garlic is fermented for 8–9 days under precisely controlled conditions of temperature (70–80 °C) and humidity (80–90 %) to produce black garlic (*9*), which is also known for its numerous health-promoting properties, including antioxidative, antiallergic, antidiabetic, anti-inflammatory and cancer-preventive effects. It also has a longer shelf life than fresh, raw garlic (*6*, *10*). During fermentation, alliinase is deactivated at a temperature range of 40–50 °C (*11*). This deactivation prevents the formation of allicin, thereby eliminating the typical pungent odour associated with fresh garlic (due to DADS), rendering black garlic free from the characteristic garlic-like smell (*12*).

Although black garlic is known for its numerous health benefits, limited research is available on its antibacterial and antifungal properties. Recent research by Mouffok *et al*. (*13*) showed the antibacterial potential of alliin-rich raw garlic extract against common human skin pathogens. However, there is a notable lack of research on the antimicrobial properties of alliin-rich black garlic extract. Furthermore, the potential correlations between the antibacterial and antifungal properties of black garlic extracts and their application in topical skin treatments remain largely unexplored.

To prevent infection and promote rapid healing, antibacterial medications such as hydrogels, sponges, ointments and nanogels are currently being widely used as effective drug delivery systems. Nanogels, in particular, offer distinct advantages over micro- and macro-scale hydrogels (*14*), including efficient drug loading, exceptional permeability across biological barriers, high water solubility, high thermal decomposition temperature, biocompatibility, and physical stability (*15*, *16*). To date, a solitary report exists on the formulation of a nanogel based on allicin-rich raw garlic extract (*17*), which is claimed to exhibit strong antifungal properties against *Candida albicans*. However, this nanogel is believed to retain the characteristic strong, pungent garlic-like odour, limiting its applicability for topical use. In contrast, a nanogel based on alliin-rich black garlic extract with similar antibacterial and antifungal potential would be more suitable for clinical use on the human skin, owing to its more acceptable sensory profile.

Carbopol® 940, a widely available polymeric carbomer known for its non-toxic and non-irritating properties (*18*), is commonly used to develop stable nanogel systems that effectively encapsulate a range of bioactive compounds. In the present study, an alliin-rich Carbopol-based antibacterial nanogel has been developed to enhance epidermal wound healing.

Therefore, the specific objectives of the current study are to evaluate the antibacterial and antifungal activity of alliin-rich black garlic extract against *Staphylococcus aureus* (ATCC), *Escherichia coli* (ATCC and MDR) and *Candida albicans* (ATCC). Additionally, based on the efficacy of the extract, the study aims to formulate a topical drug in the form of a nanogel, utilizing non-toxic, green ingredients with promising wound-healing properties that is safe for human use.

## MATERIALS AND METHODS

### Materials

Authenticated fresh garlic (*Allium sativum*) was procured from Spencer’s Retail store, Kolkata, India. Specialty chemicals such as alliin (>98 % HPLC grade) was procured from Sigma-Aldrich, Merck, Munich, Germany, l-α-phosphatidylcholine (soya lecithin 30 %) from HiMedia, Mumbai, MH, India, silica gel 60 F_254_-coated Al plates from E-Merck, Mumbai, India, low-density polyethylene (LDPE) Ziplock^®^ pouches (dimensions 25 cm×18 cm) and Al foil from Prince Plastic Pvt. Ltd., Kolkata, India; and Mueller-Hinton (MH) broth from HiMedia. American type culture collection (ATCC) strains of *Candida albicans*, *Staphylococcus aureus*, *Escherichia coli*, a multiple drug-resistant (MDR) strain of *Escherichia coli* suspensions, and antibiotic discs of Augmentin were made available by Peerless Hospital, Kolkata, India. All chemicals and reagents used in this study were of analytical reagent (AR) grade.

### Fermentation of raw garlic

Authenticated fresh garlic was procured from Spencer’s Retail store in Kolkata, India. The raw garlic samples were then placed in a rice cooker (Rize Excel 1.2 L; Kutchina, Kolkata, WB, India) under warm mode (70–80 °C) with regulated high humidity (80–90 %) for 8–9 days to undergo controlled fermentation following the method described by Lee *et al*. (*9*).

### Preparation of black garlic powder

After fermentation, the brownish-black garlic cloves were peeled and ground into a fine powder using a mortar and pestle. The powder was then wrapped in Al foil and packaged in LDPE Ziplock® pouches. The pouches were subsequently stored in a desiccator until further analysis.

### Soxhlet extraction of alliin from black garlic powder

The alliin was extracted from black garlic powder (10 g) using a Soxhlet extraction apparatus with three different green solvent systems: water, a mixture of *φ*(ethanol,water)=50 %, and ethanol, in separate batches. Conventional Soxhlet extraction with *n*-hexane or petroleum benzene was not used, as alliin is soluble in water. Each extraction was carried out for 2 h with a ratio of black garlic powder to solvent of 1:20. The extracts obtained using water, ethanol-water, and ethanol as extracting solvents were designated as BG_1_, BG_2_ and BG_3_, respectively.

### Concentration of the black garlic extracts

The black garlic extracts (BG_1_, BG_2_ and BG_3_) were concentrated using a rotary vacuum evaporator (R/160; Superfit Continental Private Ltd., Mumbai, MH, India) at 5000 Pa and a water bath temperature of (45±2) °C for 25–30 min. The extracts were further concentrated to remove solvent residues, if any, by purging with nitrogen. The yields of the extracts were determined gravimetrically. The extracts were then dissolved in distilled water and stored in amber-coloured glass vials at -18 °C for further analyses.

### Quantification of alliin using high-performance thin layer chromatography

The alliin content in the extracts (BG_E_) was quantified densitometrically with a Camag high-performance thin layer chromatography (HPTLC) unit (TLC scanner IV) at 450 nm using VisionCATS 3.0.20196.1 software (Muttenz, Switzerland) (*19*). The HPTLC analyses of BG_1_, BG_2_ and BG_3_ were conducted following the procedure by Kanaki and Rajani (*20*) with slight modifications in the mobile phase composition. The extracts in the form of 8 mm wide bands with 13.6 mm spacing between consecutive bands were applied on Al TLC plates (200 mm×100 mm) coated with silica gel 60 (F_254_) using a Camag Linomat V (Camag, Muttenz, Switzerland). Samples were separated chromatographically using *n*-butanol/acetic acid/water (6:2:2, by volume) as the mobile phase. The plate was then developed by spraying with a saturated ninhydrin reagent solution and heated at 100 °C for 5 min in a hot air oven. Visible brown colour bands of varying intensities appeared on the developed plate.

### Assessment of antimicrobial potency of standard alliin and black garlic extract

Microbial culture suspensions of *Candida albicans* (ATCC 14053), *Staphylococcus aureus* (ATCC 29213) and *Escherichia coli* (ATCC 25922 and MDR) were used (0.5 McFarland standard) to evaluate the antimicrobial potency of standard alliin and black garlic extract. The *in vitro* antimicrobial assays were conducted at Peerless Hospital, Kolkata, India, under the guidance of qualified microbiologists.

The minimum inhibitory concentrations (MIC) of standard alliin and black garlic extract were determined using the micro-broth dilution method (*21*) as described in our previous study (*22*). Black garlic extract was dissolved in distilled water to prepare a stock solution (1 mg/mL). Then, 100 μL of the stock solution of black garlic extract were added to each well of a sterile 96-well microtiter plate (0.5 McFarland was considered as the opacity standard), followed by inoculation with 10 μL of microbial culture suspension. The plate was then incubated at 37 °C for 24 h. The turbidity of the mixture in each well was assessed twice in terms of its absorbance measured at 620 nm: once immediately after the addition of the microbial culture suspension to it, and again after 24 h of incubation, using a Multiskan^TM^ FC microplate photometer (FC 357; Thermo Scientific^TM^, Waltham, MA, USA). The respective MIC values were then evaluated from the graphical plot of the concentration of standard alliin and black garlic extract (BG_E_) solution *vs* turbidity. The extract with the lowest MIC value was selected for further analysis and coded as BGE_best_.

### Compositional analysis and validation of presence of alliin in BGE_best_ using electrospray ionization-time-of-flight-mass spectrometer

The BGE_best_ was analysed using electrospray ionization-time-of-flight-mass spectrometry (ESI-TOF-MS) to determine the presence of alliin and other key bioactive compounds of garlic, namely, organosulfur bioactive compounds such as diallyl disulfide (DADS), diallyl trisulfide (DATS), methyl-allyl-disulfide and methyl-allyl-trisulfide. The spectrophotometer model Xevo-G2-Xs-QT, equipped with ADC-magnetron detector (Waters, Milford, MA, USA) was used following the method described in our previous publication (*23*). The presence of alliin in BGE_best_ was determined by comparing its mass spectrum with that of standard alliin and the presence of the organosulfur compounds was confirmed by comparing their mass spectra with those reported in the literature (*24*).

### Safety assessment of BGE_best_

Energy dispersive X-ray (EDX) analysis of BGE_best_ was carried out using a scanning electron microscope (INSPECT F50; FEI Company, Hillsboro, OR, USA) to detect toxic heavy metals such as Pb, Hg, Ti, Ni, Si, As and Mo, if any. These metals could either be inherent in raw garlic *per se* or potentially introduced during the processing stages (such as Soxhlet extraction).

### Formulation of nanogels incorporating standard alliin and BGE_best_

Two sets of homopolymeric synthetic hydrogels, one using standard alliin (as experimental control) and the other using BGE_best_, were prepared following the methods described by Iizawa *et al*. (*25*), who had formulated physically cross-linked poly (*N*-isopropylacrylamide) gel beads (*26*). Each hydrogel consisted of two phases, *i.e*. an organic and an aqueous phase. For the organic phase, a weighed amount of BGE_best_ (based on the results of MIC value) was dissolved in a mixture of dimethyl sulfoxide *w*(DMSO)=25.5 % as a solvent and *w*(soya lecithin)=0.5 % as an emulsifier to increase the strength of the gel. This mixture was vortexed for 20 min and then *w*(propylene glycol)=74 % was added as a moisturiser to improve drug permeation through animal/human skin surfaces. The resulting solution was then sonicated for 15 min at 25 °C using a bath sonicator (PCI Analytics, Mumbai, India).

For the aqueous phase, the gelling agent *w*(Carbopol^®^ 940)=2 % (HiMedia) was stirred in distilled water using a magnetic stirrer (RCT B S022; IKA, Wilmington, NC, USA) for 1 h at 1200 rpm and 80 °C. To confirm the formation of nanogel, the gel was subjected to a 2-stage homogenisation using a homogeniser (Ultra-Turrax T-50 basic homogenizer, IKA). The first stage was carried out at 6000 rpm for 30 min and the second stage at 8000 rpm for 45 min. During the second homogenisation, the organic phase (~60 mL) was added incrementally (2 mL) to the aqueous phase every 5 min. Nanogel without extract served as the experimental control. Pure standard alliin (dissolved in DMSO) was added to the aqueous phase of the nanogel developed with standard alliin (standard gel).

### Quantification of alliin in the experimental control gel and BGE_best_ gel by HPTLC

To determine the content of alliin in the prepared hydrogels by high-performance thin layer chromatography (HPTLC), 1 g of each hydrogel was dissolved in 2 mL distilled water. The solutions were vortexed followed by centrifugation (R-8C laboratory centrifuge; Remi, Mumbai, India) for 5 min at 1500×*g* and 25 °C. A volume of 30 µL of the diluted supernatant solution(s) was used to measure the alliin content according to the previously described procedure (*vide supra*).

### Physicochemical characterisation of the formulated gels

The first step was to check whether the components of the gel were actually incorporated into the matrix and whether the gels formed were actually nanogels.

Fourier transform infrared spectroscopy (FTIR) and attenuated total reflectance (ATR) analyses of the experimental control nanogel, BGE_best_ nanogel and each gel component were carried out to confirm the successful incorporation of the gel components into the hydrogel matrix. FTIR analysis of the individual constituents, *viz.* soya lecithin and Carpobol® 940 in powder form was performed using KBr pellets and an FTIR spectrometer (PerkinElmer, Waltham, MA, USA). ATR analysis of DMSO, propylene glycol, standard alliin solution, BGE_best_, experimental control hydrogel and BGE_best_ hydrogel in liquid form was conducted using a laser class I light source at a 45° angle of incidence. The FTIR and ATR spectra were analysed using the wave numbers reported by Dyer (*27*) and all constituents were found to be successfully incorporated into the gel matrices (details elaborated later).

The surface morphology and average particle size of the hydrogels were analysed using a field emission scanning electron microscope (FE-SEM) (INSPECT F50; FEI Company, Hillsboro, OR, USA) at an operating voltage of 5 kV. The gels (experimental, control and BGE_best_) were dried and gold-coated using a Q150R ES coater (Quorum Technologies Ltd., Ashford, Kent, UK). It was thereby confirmed that the hydrogels were truly nanogels (*vide infra*).

The physicochemical properties of the nanogels were analysed as follows: specific gravity using a pycnometer, pH using a digital pH meter (pc 510; Eutech Instruments, Kolkata, WB, India) and percentage mass loss on drying (at 105 °C) using the method described by Buhse *et al*. (*28*). The spreadability of the nanogels was evaluated following the method described by Ghosh *et al*. (*29*) using the following equation:


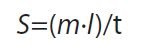
 /1/

where *S* is spreadability of the nanogel, *m* is the mass load on the cover (66 g), *l* is the length of glass slide (7.5 cm) and *t* is time.

The viscosity of the nanogels was determined using the Brookfield Digital viscometer (LVDVE230; Brookfield Engineering Company, Middleboro, MA, USA), model LVDV-E with spindle no. 4 (S_64_) at (25±1) °C. The fluid behaviour was described using the consistency index, which was determined by fitting the data into different model equations. The plot was constructed using the logarithm of shear stress and shear strain and the consistency index was calculated.

The formulated nanogels were centrifuged at 2500×*g* (TC-4815D; Eltek, Mumbai, MH, India) and phase separation studies were carried out in a water bath (Techno Lab and Instrumentations, Kolkata, WB, India) at 37 and 55 °C, respectively, following the procedures by Widodo *et al*. (*30*). The hydrophilic-lipophilic balance (HLB) values for the nanogels [HLB=20 (1-SV/AV), where SV is saponification value and AV is acid value] were determined according to the procedure described in Fennema’s Food Chemistry (*31*).

### Release profile study of BGE_best_ nanogel

The release profile of alliin from the BGE_best_ nanogel was investigated using the methodology described by Ghosh *et al*. (*29*). A mass of 0.5 g of BGE_best_ nanogel was mixed with 50 mL of phosphate-buffered saline (PBS) solution (0.1 M, pH=7.2). The mixture was continuously stirred for 2 h at 50 rpm (~34 °C) using a magnetic stirrer (RCT B S022; IKA). A volume of 3 mL of aliquot was withdrawn for 2 h at intervals of 5 min and was replaced with a similar amount of fresh PBS solution. The alliin content in the nanogel was determined using the HPTLC method described above.

### Analyses of antibacterial potency of standard alliin and nanogels containing BGE_best_

MH agar plates were prepared by dissolving 3.8 g of MH agar powder (HiMedia) in 100 mL of sterile (autoclaved) distilled water.

The zones of inhibition against uniform suspensions of *Staphylococcus aureus* (ATCC 29213) and *Escherichia coli* (ATCC 25922) and the MDR strain of *Escherichia coli* (0.5 McFarland standard) were evaluated using the Kirby-Bauer disk diffusion susceptibility test (*32*).

### Animal study on skin irritation and assessment of wound-healing property of BGE_best_ nanogel on rabbits

The skin irritation test and assessment of wound-healing efficacy of BGE_best_ nanogel were conducted at the Clinical Research Centre of Jadavpur University, in collaboration with professional experts in clinical pharmacology. For the tests, four healthy New Zealand white Albino male rabbits (R1, R2, R3 and R4), aged 6-8 months, weighing 1.5–1.8 kg were obtained from Reeta Ghosh Private Ltd., West Bengal, India. Before the experiments, the animals were placed in separate cages and allowed to get acquainted with the test environment (22 °C, 60–70 % relative humidity (RH) with a 12 h cycle of light and darkness) for 7 days. Food and drinking water were provided *ad libitum* to all animals for the entire duration of the experiment (*29*, *33*).

For future safe use in humans, the skin irritation test had to be done on the rabbits (R1, R2, R3and R4) according to the Organization for Economic Co-operation and Development (OECD) Test Guideline 404 (*34*). The skin irritation test was conducted in accordance with the Draize dermal irritation scoring system (*35*) and subsequent evaluation of the wound-healing property of the BGE_best_ nanogel was performed following the methodology described in our previous publication (*22*).

Approximately 24 h before the skin irritation test, the fur on the dorsolateral trunk of each rabbit was removed carefully and a patch of 5 cm×5 cm area was created, on which a small quantity of BGE_best_ nanogel was uniformly applied on the day of the experiment. To assess skin irritation, the rabbits (R1, R2, R3 and R4) were observed immediately after the BGE_best_ nanogel was applied and then again after 24, 48 and 72 h. All four animals were observed regularly for signs of allergic reactions, clinical toxicity, mortality or morbidity, until the completion of the experiment.

Full-thickness skin, including the *panniculus carnosus* layer, was excised from a demarcated area to create an epidermal wound of approx. 7.5±2.5 mm^2^ on each rabbit. The wound sites were gently cleansed using sterile cotton swabs. After cleansing, the following formulations were applied: a commercially available positive control gel to rabbit R1, an experimental control nanogel to R2, 2 % BGE_best_ nanogel to R3 and 4 % BGE_best_ nanogel to R4. These formulations were topically applied to the wound sites twice daily until complete epithelialization and wound closure occurred.

The physical parameters of wound healing, including wound closure, epithelialization time and scar characteristics were assessed throughout the study. Wound closure was monitored by tracing the raw wound margins onto transparent tracing paper at regular intervals from day 0 to the final day of the experiment. The wound area was calculated by counting the number of enclosed squares after transferring the traced outline of the retraced wound area on a 1 mm^2^ graph paper. The degree of wound healing was calculated as the percentage of closure of the wound area from the original one using the following formula:


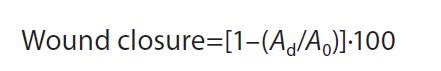
 /2/

where *A*_0_ is the wound area on day zero and *A*_d_ is the wound area on the corresponding day.

### Storage stability studies of the BGE_best_ nanogel

The storage stability of BGE_best_ nanogel was investigated by evaluating its alliin content during storage at (4±1) °C for 12 months in the dark. The amount of alliin retained in the gel was determined by HPTLC analysis at an interval of 10 days. The half-life (*t*_1/2_) of the BGE_best_ nanogel was calculated by assessing its alliin content densitometrically. The ratio of alliin content on day 0 (*w*(alliin)_0_) and on day *t* (*w*(alliin)_t_) for the gel sample was estimated and the natural logarithm of *w*(alliin)_0_/*w*(alliin)_t_ was plotted against storage time (*t*). The slope of the line (*k*) was used to obtain the half-life (*t*_1/2_=ln 2/*k*) of alliin in the BGE_best_ nanogel.

The storage stability of the BGE_best_ nanogel was evaluated by assessing its microbiological properties, primarily antibacterial activity, and visual mould growth, as well as its sensory and physicochemical properties. Two sample sets were prepared: one set was stored at (23±2) °C with 70–75 % relative humidity for 30 days (as previously described), while the other set was stored at (4±1) °C in the dark for 12 months.

### Sensory evaluation of the experimental control and BGE_best_ nanogels

The sensory evaluation of the nanogels was carried out at three stages of application on the skin: ‘picking up a sample, before rubbing it on the skin’, ‘during rubbing into the skin’ and ‘afterfeel’ when various parameters such as stiffness, grittiness, colour, odour, homogeneity, stickiness, shine, absorbance, skin feel and spreadability were evaluated (*36*). Six semi-trained panellists comprising university research scholars aged 25–34 (three males and three females) evaluated the nanogels on day 1 and day 30 of storage. The subjects were not allergic to the ingredients of the nanogel formulations and had no skin diseases. They were acquainted with the various parameters of the sensory (cosmetic) properties of the nanogels. The evaluations were carried out in a well-illuminated-cum-ventilated room. Before application, the skin surface and fingers of the panellists were wiped with clean sterile cotton. The panellists applied the nanogels on the back of their hands using ten circular movements. The responses of the panellists were recorded by rating the nanogels on a five-point hedonic scale (ranging from -2 to 2; word anchors ranging from “dislike very much” to “like very much”) according to the method described by Almeida *et al*. (*37*).

### Statistical analyses

In this study, extraction, microbiological and physicochemical assays were conducted in triplicate and the results are given as mean value±S.D. of three independent analyses of three independent batches of samples. Statistical analyses were performed using one-way analysis of variance (ANOVA). A value of p≤0.05 was used to verify the significance of all assays. All statistical analyses were conducted using IBM SPSS Statistics software v. 26 (*38*).

## RESULTS AND DISCUSSION

### Characteristics of black garlic powder and extract

The raw white garlic cloves obtained a brownish black colour after fermentation and lost most of the characteristic garlic odour. From the fermented black garlic cloves, ~95.67 % of powder with average particle size diameter *d*_p_=55 µm was obtained.

Masses of 1.27–4.96 g of Soxhlet extracts of black garlic were obtained after vacuum concentration. The lowest yield of black garlic extract (1.27 g) was obtained when ethanol was used as the extraction solvent (BG_3_), while the highest (4.96 g) yield was achieved using *φ*(ethanol,water)=50 % (BG_2_). Water used as extraction solvent yielded 4.54 g extract (BG_1_).

#### Alliin content in BG_1_ and BG_2_

**Fig. S1:**
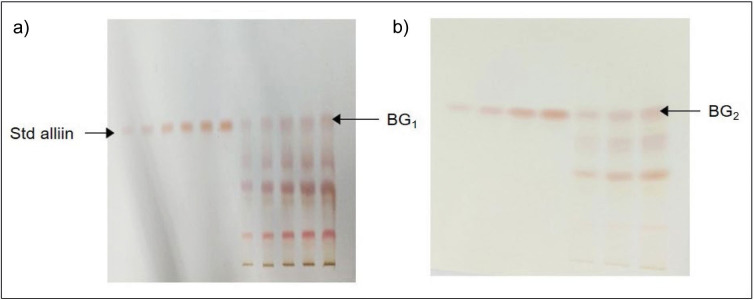
HPTLC plate for: a) BG_1_ extract, and b) BG_2_ extract with standard alliin

The alliin (R_f_ value=0.82) mass fraction in BG_1_ and BG_2_ was found to be 1.8 and 1.322 mg/g, respectively (Fig. S1). However, alliin could not be quantified in BG_3_ owing to its poor solubility in ethanol (*39*), which was used as an extraction agent (*vide supra*). BG_1_ had the highest mass fraction of alliin (p≤0.05) as alliin is highly soluble in water (*40*).

#### Antimicrobial potency of standard alliin and black garlic extract

Minimum inhibitory concentration (MIC) of standard alliin was 10, 40, 70 and 75 µg/mL for *Candida albicans* (ATCC 14053), *Staphylococcus aureus* (ATCC 29213) and *Escherichia coli* (ATCC 25922 and MDR), respectively. On average, the MIC value of BG_1_ was 15 µg/mL against the above-mentioned bacterial strains [*Staphylococcus aureus* (ATCC 29213) and *Escherichia coli* (ATCC 25922 and MDR)] and that of BG_2_ was 25 µg/mL for the same. The MIC values of both BG_1_ and BG_2_ were much higher (>90 µg/mL) against the above-mentioned fungal strain *Candida albicans* (ATCC 14053). Based on these results, it can be inferred that although standard alliin shows potent antimicrobial activity, the antibacterial properties of black garlic extract were stronger than its antifungal properties. Based on the results of the one-way ANOVA for the MIC values of standard alliin and black garlic extract (BG_1_ and BG_2_), it can be concluded that BG_1_ had a significantly lower (p≤0.05) MIC value, indicating its higher ability to inhibit bacterial growth than BG_2_. Therefore, BG_1_ was designated as BGE_best_ and used to produce the nanogel.

To the best of our knowledge, there is no available literature on alliin content of white and black garlic and none on antimicrobial potency of alliin-rich black garlic extract to allow comparison of our findings.

#### Composition and safety of BGE_best_

Based on the ESI-TOF-MS spectrum of the pure standard of alliin (Table 1), it was confirmed that the alliin mass fraction quantified by HPTLC in BGE_best_ corresponds unequivocally to the target biomolecule, alliin. A similar ESI spectrum for alliin was reported by Tran *et al*. (*41*).

**Table 1:** Results of ESI-TOF-MS analysis of alliin standard and the alliin and additional organosulfur bioactive compounds present in black garlic extract (BGE_best_)

Compound	*m*/*z*	Relative absorbance/%
Alliin standard		
S-allyl-l-cysteine (alliin) C_6_H_11_NO_2_S (162.22)	162.817	45
S-allyl-l-cysteine (alliin) C_6_H_11_NO_2_S+H (162.22+1)	163.062 163.102 163.154 163.580	100 95 85 80
BGE_best_		
S-allyl-l-cysteine (alliin) C_6_H_11_NO_2_S (162.22)	162.62	90
S-allyl-l-cysteine (alliin) C_6_H_11_NO_2_S-H (162.22-1)	161.348 161.120 161.151	100 98 85
S-allyl-l-cysteine (alliin) C_6_H_11_NO_2_S–H_2_O+H+Na (162.06–18+1+23)	168.211 168.243	100 70
Di-allyl-disulfide (DADS) C_6_H_10_S_2_+Na+H (146.022+23+1)	170.509 170.450	100 70
Di-allyl-trisulfide (DATS) C_6_H_10_S_3_ (177.194)	177.161 177.210 177.137	100 95 65
Methyl-allyl-disulfide (MADS) C_4_H_8_S_2_+H (120.007+1)	121.471 121.459 120.869 121.312	100 85 80 70
Methyl-allyl-trisulfide (MATS) C_4_H_8_S_3_ (151.979)	151.916 151.725 151.657 151.616	100 95 65 60

From the *m*/*z* values ranging from 143 to 180 in the ESI-TOF-MS spectra (Table 1) of BGE_best_ (*24*), several additional bioactive organosulfur compounds such as di-allyl-disulfide, di-allyl-trisulfide, methyl-allyl-disulfide and methyl-allyl-trisulfide were tentatively identified. These organosulfur compounds present in black garlic extract are reportedly non-toxic (*6*) and have antibacterial and antifungal properties (*4*, *5*).

The EDX spectra of BGE_best_ (data not shown) did not reveal peaks of heavy metals, thereby confirming the absence of heavy metal contaminants in the formulation. These findings confirm that the extract is non-toxic and therefore safe for the formulation of a topical antibacterial nanogel for potential clinical application.

### Physicochemical properties of the nanogels (experimental control and BGE_best_)

The standard alliin-based nanogel was odourless and clear in appearance, while the BGE_best_ nanogel had a pale-yellow colour and translucent appearance with a slight garlic characteristic odour.

#### FTIR and ATR spectra of the nanogels

FTIR and ATR spectra were recorded for Carbopol^®^ 940, DMSO, propylene glycol, soya lecithin (30 %), standard alliin, BGE_best_, experimental control nanogel and BGE_best_ nanogel. The transmission (*T*/%) peaks of the BGE_best_ nanogel were similar to those of the standard alliin solution. The transmission peaks of nanogels matched those of the individual components in the formulation. FTIR and ATR analyses confirmed that the Soxhlet extract (BG_1_), which is rich in alliin, had been effectively incorporated into the nanogel matrix.

Table 2 shows the functional groups and the absorbance bands prominently present in the FTIR spectra of the analysed samples. The spectra showed the presence of O-H stretching (3628, 3348  cm^-1^), C-H stretching (2853 cm^-1^), C=C alkene stretching (1651 cm^-1^), C-O-C stretching (1249, 1225 cm^-1^) and C-F stretching (1044, 1016 cm^-1^) frequencies, which confirmed the presence of standard alliin and other ingredients in the BGE_best_ nanogel.

**Table 2:** FTIR spectra of Carbopol^®^ 940, DMSO, propylene glycol, soya lecithin (30 %), standard alliin, BGE_best_, experimental control nanogel and black garlic extract-based (BGE_best_) nanogel

Wavenumber/cm^-1^							
Carbopol^®^ 940	DMSO	Propyleneglycol	Soya lecithin (30 %)	Standard alliin	BGE_best_	Experimental control nanogel	BGE_best_ nanogel
3118: O-H stretching	3442: O-H stretching	3307: O-H stretching	3445: O-H stretching	3331: O-H stretching	3259: O-H stretching	3272: O-H stretching	3628, 3348: O-H stretching
1713: C=O ketone stretching	2995: =C-H stretching	2969, 2929, 2875: carboxylic acid OH stretching	2925: C-H stretching	2920, 2836: C-H stretching	2948: C-H stretching	2130: C-H aldehydic stretching	2853: C-H stretching
1454: CH_3_ stretching	2912: C-H stretching	1455, 1411: C=C aromatic	1651,164, 1634: C-C multiple bonds stretching	1646: C=C alkene	1641, 1405: C=C alkene stretching	1635, 1420: C=C aromatic	1651: C=C alkene stretching
1114, 117, 1247: C-O-C stretching	1435: C=C aromatic	1375,1331: CH_3_ stretching	1732: ester stretching	1575, 1540, 1452, 1417: C=C aromatic	1118, 1013: C-O-C stretching	1077, 1039: C-O-C stretching	1249,1225: C-O-C stretching
801,648: C-Cl stretching	1040, 1017: C-F stretching	1232, 1135, 1076, 1036: C-O-C stretching	1466: C-H_2_ bending	1015: C-F stretching	n.d.	n.d.	1044, 1016: C-F stretching
n.d.	760: C-Cl stretching	n.d.	1377: CH_2_-O-P-O	n.d.	n.d.	n.d.	n.d.
n.d.	n.d.	n.d.	1226,108: C-N	n.d.	n.d.	n.d.	n.d.

n.d.=not detected

#### Microstructure of the experimental control and BGE_best_ nanogels

**Fig. 1:**
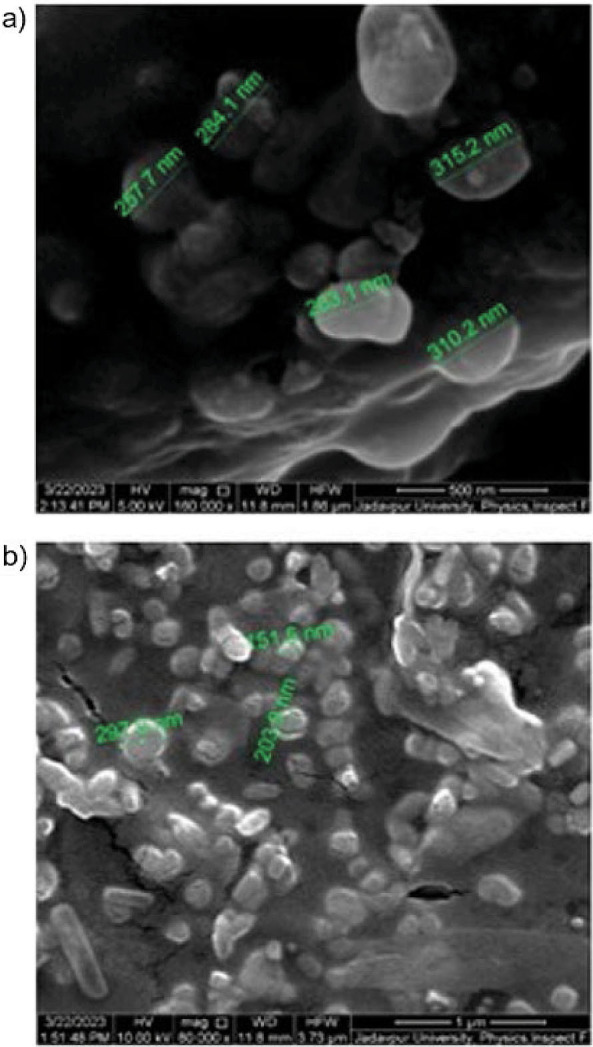
FE-SEM images of: a) experimental control nanogel and b) black garlic extract-based (BGE_best_) nanogel

The FE-SEM analyses showed that the experimental control and BGE_best_ nanogels had smooth spherical surfaces. The average particle size of the experimental control and BGE_best_ nanogels was 286.06 and 217.77 nm, respectively (Fig. 1), which corresponds to the dimensions of other reported nanogels, such as that of kappa-carrageenan/chitosan (*42*). The findings show that the average particle size of the formulated gel was in the nanometer range and thus the gel can be classified as a nanogel.

#### Other physicochemical properties of the nanogels

Table 3 shows the results of physicochemical analyses of the formulated topical nanogels. The pH values of 6.90±0.02 and 6.82±0.01 of the control and BGE_best_ nanogels, respectively, were well within the range suitable for use as a topical medication compatible with the human skin (*43*). Similar pH values were reported by Wadile *et al*. (*44*) for their nanogel incorporated with itraconazole nanoparticles (pH=6.8) and by Ali *et al*. (*45*), who reported a pH=6.87 of lidocaine wound-healing nanogel. The specific gravity of the control was 1.038±0.005 and of BGE_best_ nanogel 1.021±0.005 and the mass loss on drying was (2.31±0.01) % for the control and (1.5±0.01) % for the BGE_best_ nanogel. The spreadability of nanogel, a crucial aspect that affects its viscosity and cosmetic (sensory) acceptability (*46*), was (15.92±0.02) g/(cm·s) for the control and (12.41±0.02) g/(cm·s) for the BGE_best_ nanogel. A similar value for spreadability was reported by Inamdar *et al*. (*47*) for nanogel loaded with β-sitosterol as its bioactive component.

**Table 3:** Results of physicochemical analyses of the newly developed topical nanogels

Property	Experimental control nanogel	BGE_best_ nanogel
Appearance	Clear	Pale yellow
Specific gravity	1.038±0.005	1.021±0.005
pH at 25 °C	6.90±0.02	6.82±0.01
*m*(loss on drying)/%	2.31±0.01	1.50±0.01
Spreadability/(g/(cm·s))	15.92±0.02	12.41±0.02
Centrifuge test	No phase separation	No phase separation
Phase separation at 37 °C	No phase separation	No phase separation
Phase separation at 55 °C	No phase separation	No phase separation
HLB	17.0±0.2	18.6±0.2
*η*/(mPa∙s)	18.92±0.02	23.03±0.02
*d*(particle size)/nm	286.06	217.77

Values are reported as mean value±standard deviation (S.D.). HLB=hydrophilic-lipophilic balance, BGE_best_ nanogel=black garlic extract-based nanogel

**Fig. S2:**
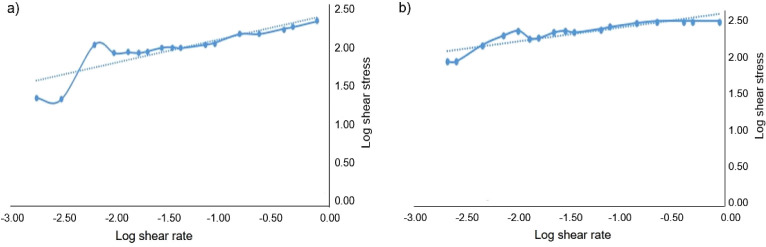
Viscosity graph for log shear stress *vs* log shear rate using modified Casson equation for: a) experimental control nanogel and b) black garlic extract-based (BGE_best_) nanogel

The plot of log shear stress *vs* log shear rate (Fig. S2) showed that the nanogels are non-Newtonian fluids. Model fitting revealed that viscosity data best fit the modified Casson equation:


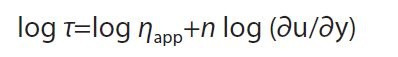
 /3/

where *τ* is the shear stress (N/m^2^), ∂u/∂y is a shear rate (s^-1^), *η*_app_ is apparent viscosity (mPa∙s) and *n* is flow behaviour index.

The flow index (*n*<1) using the above equation indicates that the nanogels show pseudoplastic flow behaviour. Eq. 3 shows that the apparent viscosity of the control nanogel is (18.92±0.02) mPa∙s and that of the BGE_best_ nanogel is (23.03±0.02) mPa∙s. Since nanogels have shear thinning behaviour and appreciable spreadability, they are advantageous for effortless topical application. A similar flow behaviour was reported by Agarwal *et al*. (*48*) for a semi-herbal nanogel containing clindamycin phosphate and aloe vera. No phase separation after centrifugation at 37 and 55 °C indicated that the formulation was properly homogenised. HLB value for the experimental control nanogel was 17.0±0.2 and that for the BGE_best_ nanogel was 18.6±0.2, indicating that the formulation was hydrophilic (*31*).

### In vitro release profile of BGE_best_ nanogel

The BGE_best_ nanogel showed a 75 % burst release of alliin within 5 min, indicating a high release rate. This rapid release is advantageous for immediate drug delivery with topical application, making it beneficial for wound healing (*49*). There are few reports on *in vitro* studies on the release kinetics of topical hydrogels/nanogels; however, a similar burst release of dexamethasone from polylactic-co-glycolic acid (PLGA) microspheres embedded in polyvinyl alcohol (PVA) hydrogel in the submicron range was observed by Gu and Burgess (*50*).

### Antibacterial potency of the formulated nanogels

**Fig. S3:**
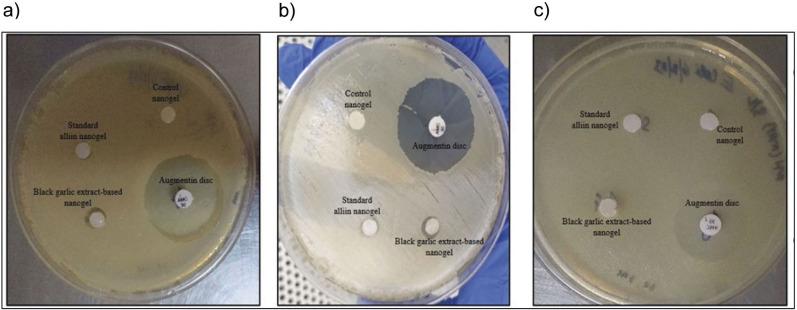
Zones of inhibition of standard alliin-based nanogel and black garlic extract-based (BGE_best_) nanogel against: a) *Staphylococcus aureus* (ATCC), b) *Escherichia coli* (ATCC) and c) *Escherichia coli* (MDR). MDR=multiple drug-resistant

The zone of inhibition of standard alliin-based nanogel was 7 mm against *Staphylococcus aureus* (ATCC 29213) and that of BGE_best_ nanogel was 10 mm. The zone of inhibition of the standard alliin-based nanogel against *Escherichia coli* (ATCC 25922) was 7 mm and 8 mm of BGE_best_ nanogel. Inhibition zones of standard alliin-based nanogel and BGE_best_ nanogel against *Escherichia coli* MDR strain was 6 and 7 mm, respectively (Fig. S3). The one-way ANOVA of the diameters of the zones of inhibition of standard alliin-based nanogel and the extract-based nanogels leads to a conclusion that the BGE_best_ nanogel had significantly greater (p≤0.05) antibacterial potency than the standard alliin-based nanogel, which renders the formulated nanogel a potent therapeutic formulation for topical wound-healing.

### Skin irritation and wound-healing properties in rabbits

#### Skin irritation property of BGE_best_ nanogel on rabbits

**Fig. S4:**
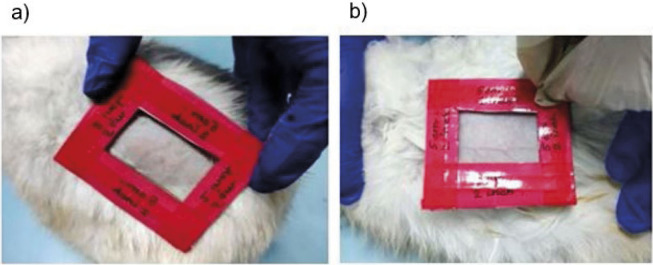
Skin irritation test of black garlic extract-based (BGE_best_) nanogel on New Zealand white rabbits: a) demarcated area on dorsal skin immediately after application (0 h) and b) observation 72 h after nanogel was applied

The primary irritation index (PII) scores of the nanogel for erythema and oedema were zero after 72 h, which suggested that there were no indications of skin irritation at the test site during the period of study (Fig. S4). After the application of the nanogel to the skin surface, the rabbits showed no clinical signs of toxicity or irritation. Therefore, it can be concluded that the BGE_best_ nanogel is a safe, non-toxic formulation with no adverse irritant effects [PII (0/36=0)] when applied to rabbit skin. This suggests that the nanogel can safely be used topically to evaluate its efficacy in improving epidermal wound-healing.

#### Wound-healing efficacy of BGE_best_ nanogel

**Fig. S5:**
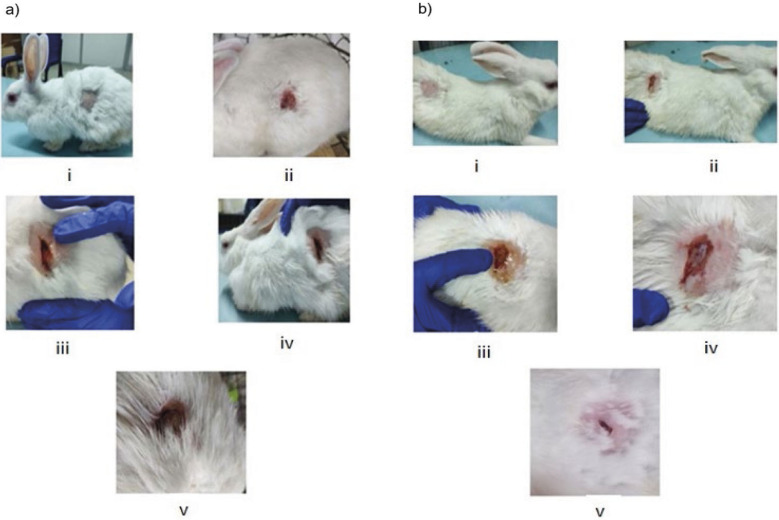
Wound-healing test on: a) rabbit R3 using black garlic extract-based (BGE_best_) nanogel (2 %): *i*) before wound creation, *ii*) created wound, *iii*) observation on day 0 after the application, *iv*) initiation of tissue epithelization on day 3, *v*) wound closure on day 6, and b) rabbit R4 using BGE_best_ nanogel (4 %) *i*) before wound creation, *ii*) created wound, *iii*) observation on day 0 after the application, *iv*) initiation of tissue epithelization on day 2, *v*) wound closure on day 6

Fig. S5a and Fig. S5b show the epidermal wound-healing experiment using BGE_best_ nanogels (2 and 4 %) on rabbits R3 and R4, respectively (Table 4). Following topical application, tissue epithelization surrounding the injuries (in R3 and R4) commenced on day 2. By the end of day 6, 67 and 77 % of wounds healed on R3 and R4, respectively, which was found to be almost similar to the wound-healing efficacy of the positive control gel, *i.e.* 86 % wound closure by day 6 on R1. In contrast, the control nanogel showed no signs of wound closure on R2, confirming that the experimental control nanogel alone did not contribute to wound healing, thereby validating the wound-healing properties of BGE_best_ nanogel.

**Table 4:** The wound-healing area on each day after the application of positive control gel, experimental control nanogel and black garlic extract-based (BGE_best_) nanogel

*t*/day	*A*/mm^2^			
	R1	R2	R3	R4
0	7.5	4.5	4.5	6.5
1	5	4.5	4	6
2	3	4.5	3.5	4.5
3	1	n.d.	2.5	3.5
4	n.d.	n.d.	2	2.5
5	n.d.	n.d.	1.5	1.5
6	n.d.	n.d.	1.5	1.5

R1=positive control gel, R2=experimental control nanogel, R3=nanogel with *w*(BGE_best_)=2 %, R4=nanogel with *w*(BGE_best_)=4 %; n.d.=not determinable

Results of the one-way ANOVA analysis of wound-healing areas after the application of positive control gel, experimental control nanogel, and 2 and 4 % BGE_best_ nanogels in the respective animal groups on a daily basis showed that the wound-healing efficacy of the 4 % BGE_best_ nanogel (in R4 group) was significantly higher (p≤0.05) than that of the 2 % BGE_best_ nanogel (in R3 group). The healing process in R4 group was notably accelerated. In addition, the wound sites were effectively protected from any external infection by applying 2 % BGE_best_ nanogel in R3 and 4 % BGE_best_ nanogel in R4. By day 7, the wound of R4 completely healed with no scarring, confirming that the 4 % BGE_best_ nanogel is a promising topical antibacterial nanogel with enhanced wound-healing properties.

A study reported by Ahmed *et al*. (*51*) showed similar findings of accelerated wound-healing in rabbits using a hydrogel formulated with the herb *Centella asiatica*. At the end of day 5 of treatment, wounds treated with the hydrogel showed complete wound closure with the formation of a thin epidermal layer; and by the end of day 9, all wounds were healed with a concomitant thickening of the epidermis layer. The wound reduction in rabbits treated with *Centella asiatica* hydrogel was approx. 40 % higher than the untreated wounds in the control group (*51*).

### Stability of the BGE_best_ nanogel

The half-life (*t*_1/2_) of BGE_best_ nanogel was approx. 193 days, although the alliin content remained above its MIC until 120 days of storage. After this period, the alliin content gradually decreased below the MIC value (15 µg/mL). Therefore, it is recommended to store the BGE_best_ nanogel at (4±1) °C and use it within 120 days to ensure optimal effectiveness.

### Sensory attributes of the experimental control and BGE_best_ nanogels

**Fig. 2:**
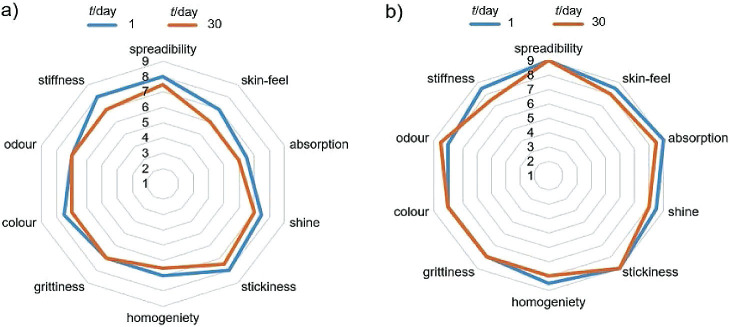
Radar plot of hedonic scores obtained by sensory analyses of: a) experimental control nanogel and b) nanogel based on black garlic extract (BGE_best_) on days 1 and 30

During sensory evaluation of the nanogels, the panel preferred the non-sticky consistency and shiny appearance of BGE_best_ nanogel. Both the experimental control and BGE_best_ nanogel were non-irritable, easily spreadable, moderately slimy and very shiny with good to moderate absorption capacity (Fig. 2). The findings suggested that the panel validated the formulation as a topical nanogel with moisturizing property. The radar plot analysis of the hedonic scores obtained using the 9-point hedonic scale showed that BGE_best_ nanogel had the highest score (9) for spreadability and absorption, *vis-à-vis* 8 and 6, respectively, for the control set of nanogel. BGE_best_ nanogel also received higher scores than other samples for other cosmetic characteristics such as colour, odour, homogeneity and texture properties (stickiness and skin feel).

The shear thinning property of BGE_best_ nanogel made it more spreadable, which led to good absorption and great primary and secondary skin feel, as is evident (Fig. 2) from its sensory scores (*52*). The nanogel could be spread evenly on the skin surface without being highly adhesive or tacky (*30*). The safety of the nanogel was further validated by the skin irritation test on rabbits as discussed above. The present study has conclusively demonstrated the potential of nanogel based on black garlic extract for topical application on human skin.

### Comparison of the newly formulated nanogel with reported gels having wound-healing properties

These attributes align well with pharmaceutical recommendations for effective topical delivery of active ingredients through the human skin, as reported by Kulawik-Pióro *et al*. (*53*), who investigated the quality of barrier creams composed of International Nomenclature of Cosmetic Ingredients (INCI), namely, aqua, glycerine, sodium silicate, sodium palm kernelate, ceteareth-5, sodium tallowate, cera alba, paraffin, parfum, *etc*. with similar attributes.

A commercially available and commonly prescribed gel used for wound-healing is Hydroheal AM gel, which is formulated with non-green, toxic ingredients (colloidal silver) and is known to cause argyria, a permanent bluish-grey skin discolouration, when applied to human skin for prolonged periods. It can also interfere with the absorption of drugs and lead to potential problems with kidney, liver and the nervous system (*54*). Moreover, since colloidal silver is one of the main constituents of this gel, it cannot be used to heal open wounds because the silver oxidizes readily in atmospheric air and produces silver oxide that may cause greyish-black discolouration when applied to the skin (*55*, *56*). Thus, to overcome this disadvantage, it is advisable to use the commercial gel only before the epidermal/dermal wound dressing and then the wound must be bandaged to prevent direct contact with air.

On the contrary, the newly formulated BGE_best_ nanogel contains a safe, non-toxic organosulfur compound with alliin-rich black garlic extract and non-toxic, green ingredients completely safe for human application against common potent skin pathogens and for long-term usage to heal open epidermal wounds with promising efficacy.

## CONCLUSIONS

This study is the first to describe the antibacterial activity of a safe, green nanogel loaded with non-toxic alliin-rich black garlic extract. The obtained yellow nanogel showed significant antibacterial activity against the common bacterial pathogens *Staphylococcus aureus* and *Escherichia coli*. The nanogel formulation produced homogeneous, spherical, lump-free particles in the nanometer range with a smooth surface. With a pH range of 6.82 to 6.90, black garlic extract obtained using water as a solvent (BG_1_) performed much better than the extract obtained using etanol/water mixture (BG_2_) and had much higher drug content and minimum inhibitory concentration value. The formulated nanogel can be used for topical application with ease owing to its excellent spreadability and viscosity. The spreadability value of the nanogel with the lowest MIC value (BGE_best_) and its pseudoplastic behaviour further support its use for topical applications. FTIR and ATR analyses confirmed the successful integration of alliin-rich BGE_best_ into the nanogel matrix with its other components. Skin irritation and epidermal wound-healing studies with the BGE_best_ nanogel in rabbits showed positive effects. The control gel did not promote wound healing, while the addition of the extract accelerated the healing process. The study also demonstrated that the percentage of wound closure increased with the increase in the content of the active ingredient (BGE_best_). Short-term stability, skin irritation and wound-healing studies of the formulation along with positive panellist feedback confirm the viability and acceptance of the nanogel formulation for topical use.

Future investigation of potential applications of the nanogel should explore its effectiveness against other severe bacterial skin infections. Additionally, other types of hydrogels can be formulated using natural polymers, such as chitosan, pectin, gelatin and agar (alongside synthetic polymers like Carbopol® 940) to create safe nanogels suitable for both animal and human use and environmental sustainability. There is also significant potential to use other pharmacological and biological properties of black garlic extract, such as its antioxidant, anti-inflammatory and anti-allergic effects, for the formulation of nanogels, hydrogels and ointments. Further investigation into the underlying mechanisms of these findings could provide valuable insights into its wound-healing ability.
